# Supplementary material for: Functional immune responses against SARS-CoV-2 variants of concern after fourth COVID-19 vaccine dose or infection in patients with blood cancer
Source: Cell Rep Med. 2022 Sep 27;3(10):100781. doi: 10.1016/j.xcrm.2022.100781 (PMC9513326; doi:10.1016/j.xcrm.2022.100781)
Supplement: Document S1. Tables S1 and S2 [file mmc1.pdf]

Cell Reports Medicine, Volume *3*

Supplemental information

Functional immune responses against SARS-CoV-2 variants of concern after fourth COVID-19 vaccine dose or infection in patients with blood cancer

Annika Fendler, Scott T.C. Shepherd, Lewis Au, Mary Wu, Ruth Harvey, Katalin A. Wilkinson, Andreas M. Schmitt, Zayd Tippu, Benjamin Shum, Sheima Farag, Aljosja Rogiers, Eleanor Carlyle, Kim Edmonds, Lyra Del Rosario, Karla Lingard, Mary Mangwende, Lucy Holt, Hamid Ahmod, Justine Korteweg, Tara Foley, Taja Barber, Andrea Emslie-Henry, Niamh Caulfield-Lynch, Fiona Byrne, Daqi Deng, Svend Kjaer, Ok-Ryul Song, Christophe J. Queval, Caitlin Kavanagh, Emma C. Wall, Edward J. Carr, Simon Caidan, Mike Gavrielides, James I. MacRae, Gavin Kelly, Kema Peat, Denise Kelly, Aida Murra, Kayleigh Kelly, Molly O'Flaherty, Robyn L. Shea, Gail Gardner, Darren Murray, Sanjay Popat, Nadia Yousaf, Shaman Jhanji, Kate Tatham, David Cunningham, Nicholas Van As, Kate Young, Andrew J.S. Furness, Lisa Pickering, Rupert Beale, Charles Swanton, Sonia Gandhi, Steve Gamblin, David L.V. Bauer, George Kassiotis, Michael Howell, Emma Nicholson, Susanna Walker, Robert J. Wilkinson, James Larkin, and Samra Turajlic

Table S1: Ordinal regression model of factors associated with Omicron BA.1 NAbT responses after 4 vaccine doses

	Ordinal regression model				ANOVA	
Factor	Coefficient	S.E.	Wald Z	P-value	Chi-Square	p-value
**Vaccine type (first and second dose)**					0.13	0.72
BNT162b2 (vs ChAdOx1)	-0.17	0.48	-0.35	0.72		
**Previous infection**					3.44	0.06
SarS-CoV-2 infection before second vaccine dose	1.40	0.76	1.86	0.06		
**Diagnosis (vs acute leukaemia)**					3.52	0.47
Chronic lymphocytic leukaemia	0.34	0.98	0.35	0.72		
Myelodysplastic syndrome	-0.95	1.41	-0.67	0.50		
Myeloma	0.11	0.98	0.12	0.91		
Lymphoma	1.31	1.04	1.26	0.21		
**Anti-cancer therapy**						
B cell-depleting therapy	-3.72	0.85	-4.35	<0.0001	18.89	<.0001
Chemo- or targeted therapy	-0.15	0.64	-0.24	0.81	0.06	0.81

NAbT were binned in undetected/low (=< 40), moderate (>40-256), or high (>256) †For anti-cancer therapy indicated treatment was tested for patients who received the treatment vs patients not receiving that treatment. BTKi, Bruton's tyrosine kinase inhibitor.

Table S2: Ordinal regression model of factors associated with Omicron BA.2 NAbT responses after 4 vaccine doses

	Ordinal regression model				ANOVA	
Factor	Coefficient	S.E.	Wald Z	P-value	Chi-Square	p-value
**Vaccine type (first and second dose)**					0.00	0.98
BNT162b2 (vs ChAdOx1)	-0.01	0.51	-0.02	0.98		
**Previous infection**					1.01	0.32
SarS-CoV-2 infection before second vaccine dose	0.76	0.76	1.00	0.32		
**Diagnosis (vs acute leukaemia)**					3.5	0.48
Chronic lymphocytic leukaemia	0.85	1.00	0.85	0.40		
Myelodysplastic syndrome	1.17	1.55	0.75	0.45		
Myeloma	1.52	1.04	1.46	0.14		
Lymphoma	1.57	1.06	1.48	0.14		
**Anti-cancer therapy**						
B cell-depleting therapy	-3.01	0.81	-3.73	0.0002	13.88	0.0002
Chemo- or targeted therapy	-0.62	0.73	-0.85	0.40	0.72	0.40

NAbT were binned in undetected/low (=< 40), moderate (>40-256), or high (>256) †For anti-cancer therapy indicated treatment was tested for patients who received the treatment vs patients not receiving that treatment. BTKi, Bruton's tyrosine kinase inhibitor.
