# Supplementary material for: Functional immune responses against SARS-CoV-2 variants of concern after fourth COVID-19 vaccine dose or infection in patients with blood cancer
Source: Cell Rep Med. 2022 Sep 27;3(10):100781. doi: 10.1016/j.xcrm.2022.100781 (PMC9513326; doi:10.1016/j.xcrm.2022.100781)
Supplement: Document S2. Article plus supplemental information [file mmc2.pdf]

Cell Reports Medicine

Report

Functional immune responses against SARS-CoV-2 variants of concern after fourth COVID-19 vaccine dose or infection in patients with blood cancer

Graphical abstract

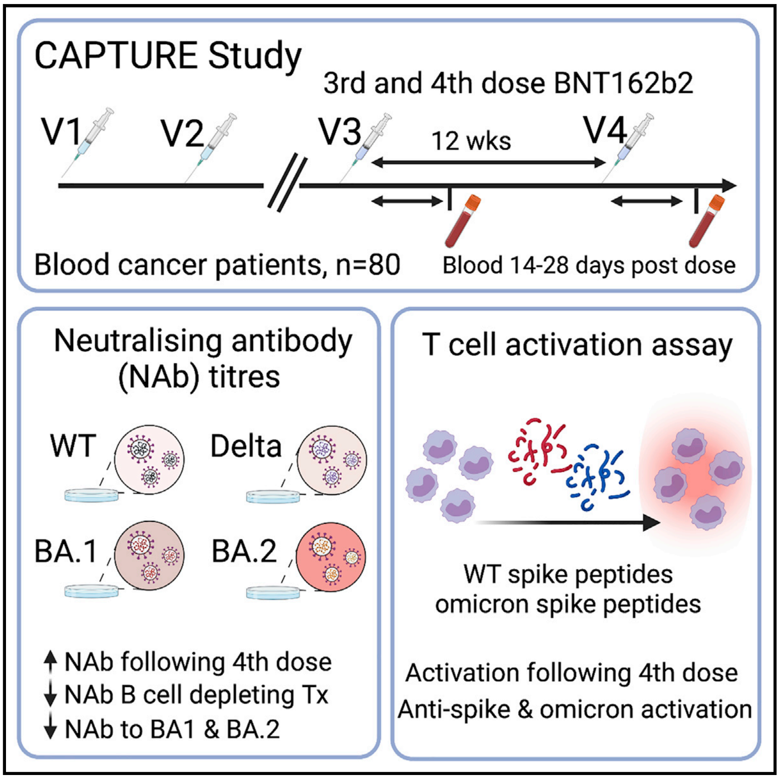

Highlights

- Neutralizing antibody (NAb) responses following fourth COVID-19 vaccine dose
- SARS-CoV-2-specific T cell responses increased following fourth COVID-19 dose
- NAb responses reduced to Omicron BA.1 and BA.2 lineages compared to wild type
- B cell-depleting therapy within 12 months associated with undetectable NAbs

Authors

Annika Fendler, Scott T.C. Shepherd, Lewis Au, ..., Robert J. Wilkinson, James Larkin, Samra Turajlic

Correspondence

samra.turajlic@crick.ac.uk

In brief

Fendler et al. evaluate neutralizing antibody (Nab) and cellular responses to a fourth COVID-19 vaccination in patients with blood cancer. The proportion of patients with detectable NAb and T cell responses increased after the fourth vaccine dose; however, some had suboptimal NAb responses, in particular, those receiving B cell-depleting therapies.

Fendler et al., 2022, Cell Reports Medicine *3*, 100781
October 18, 2022 © 2022 The Author(s).
https://doi.org/10.1016/j.xcrm.2022.100781

CellPress

Cell Reports Medicine

CellPress
OPEN ACCESS

Report

Functional immune responses against SARS-CoV-2 variants of concern after fourth COVID-19 vaccine dose or infection in patients with blood cancer

Annika Fendler,[1,34] Scott T.C. Shepherd,[1,2,34] Lewis Au,[1,2,34] Mary Wu,[3,34] Ruth Harvey,[4,34] Katalin A. Wilkinson,[5,6,34] Andreas M. Schmitt,[2] Zayd Tippu,[1,2] Benjamin Shum,[1,2] Sheima Farag,[2] Aljosja Rogiers,[2] Eleanor Carlyle,[2] Kim Edmonds,[2] Lyra Del Rosario,[2] Karla Lingard,[2] Mary Mangwende,[2] Lucy Holt,[2] Hamid Ahmod,[2] Justine Korteweg,[2] Tara Foley,[2] Taja Barber,[1] Andrea Emslie-Henry,[1] Niamh Caulfield-Lynch,[1] Fiona Byrne,[1] Daqi Deng,[1] Svend Kjaer,[7] Ok-Ryul Song,[8] Christophe J. Queval,[8] Caitlin Kavanagh,[3] Emma C. Wall,[8,9] Edward J. Carr,[10] Simon Caidan,[11]

(Author list continued on next page)

[1]Cancer Dynamics Laboratory, The Francis Crick Institute, London NW1 1AT, UK
[2]Skin and Renal Units, The Royal Marsden NHS Foundation Trust, London SW3 6JJ, UK
[3]COVID Surveillance Unit, The Francis Crick Institute, London NW1 1AT, UK
[4]Worldwide Influenza Centre, The Francis Crick Institute, London NW1 1AT, UK
[5]Tuberculosis Laboratory, The Francis Crick Institute, London NW1 1AT, UK
[6]Wellcome Center for Infectious Disease Research in Africa, University of Cape Town, Observatory 7925, Republic of South Africa
[7]Structural Biology Scientific Technology Platform, The Francis Crick Institute, London NW1 1AT, UK
[8]High Throughput Screening Laboratory, The Francis Crick Institute, London NW1 1AT, UK
[9]University College London Hospitals NHS Foundation Trust Biomedical Research Centre, London WC1E 6BT, UK
[10]Cell Biology of Infection Laboratory, The Francis Crick Institute, London NW1 1AT, UK
[11]Safety, Health & Sustainability, The Francis Crick Institute, London NW1 1AT, UK
[12]Scientific Computing Scientific Technology Platform, The Francis Crick Institute, London NW1 1AT, UK
[13]Metabolomics Scientific Technology Platform, The Francis Crick Institute, London NW1 1AT, UK
[14]Department of Bioinformatics and Biostatistics, The Francis Crick Institute, London, UK
[15]Department of Pathology, The Royal Marsden NHS Foundation Trust, London NW1 1AT, UK
[16]Translational Cancer Biochemistry Laboratory, The Institute of Cancer Research, London SW7 3RP, UK
[17]Lung Unit, The Royal Marsden NHS Foundation Trust, London SW3 6JJ, UK
[18]Acute Oncology Service, The Royal Marsden NHS Foundation Trust, London SW3 6JJ, UK
[19]Anaesthetics, Perioperative Medicine and Pain Department, The Royal Marsden NHS Foundation Trust, London SW3 6JJ, UK
[20]Gastrointestinal Unit, The Royal Marsden NHS Foundation Trust, Sutton SM2 5PT, UK
[21]Clincal Oncology Unit, The Royal Marsden NHS Foundation Trust, London NW1 1AT, UK

(Affiliations continued on next page)

SUMMARY

Patients with blood cancer continue to have a greater risk of inadequate immune responses following three COVID-19 vaccine doses and risk of severe COVID-19 disease. In the context of the CAPTURE study (NCT03226886), we report immune responses in 80 patients with blood cancer who received a fourth dose of BNT162b2. We measured neutralizing antibody titers (NAbTs) using a live virus microneutralization assay against wild-type (WT), Delta, and Omicron BA.1 and BA.2 and T cell responses against WT and Omicron BA.1 using an activation-induced marker (AIM) assay. The proportion of patients with detectable NAb titers and T cell responses after the fourth vaccine dose increased compared with that after the third vaccine dose. Patients who received B cell-depleting therapies within the 12 months before vaccination have the greatest risk of not having detectable NAbT. In addition, we report immune responses in 57 patients with breakthrough infections after vaccination.

INTRODUCTION

A third COVID-19 vaccine dose induces functional immune responses in most patients with cancer, including neutralizing antibodies (NAbs) against variants of concern (VOCs) and T cell responses. However, some patients with blood cancer, especially those receiving B cell-depleting therapies, have inadequate immune responses even after a third dose[1,2] and may, in turn, have a higher risk of breakthrough infection. Regarding the Omicron BA.1 variant, NAb response increased after three doses

This is an open access article under the CC BY license (http://creativecommons.org/licenses/by/4.0/).

Mike Gavrielides,[12] James I. MacRae,[13] Gavin Kelly,[14] Kema Peat,[2] Denise Kelly,[2] Aida Murra,[2] Kayleigh Kelly,[2] Molly O'Flaherty,[2] Robyn L. Shea,[15,16] Gail Gardner,[15] Darren Murray,[15] Sanjay Popat,[17] Nadia Yousaf,[17,18] Shaman Jhanji,[19] Kate Tatham,[19] David Cunningham,[20] Nicholas Van As,[21] Kate Young,[2] Andrew J.S. Furness,[2] Lisa Pickering,[2] Rupert Beale,[10,22] Charles Swanton,[23,24] Sonia Gandhi,[25,26] Steve Gamblin,[27] David L.V. Bauer,[28] George Kassiotis,[29] Michael Howell,[8] Emma Nicholson,[30,31] Susanna Walker,[19] Robert J. Wilkinson,[5,6,32] James Larkin,[2,33] and Samra Turajlic[1,2,33,35,*]
[22]Division of Medicine, University College London, London NW1 2PG, UK
[23]Cancer Evolution and Genome Instability Laboratory, The Francis Crick Institute, London NW1 1AT, UK
[24]University College London Cancer Institute, London WC1E 6DD, UK
[25]Neurodegeneration Biology Laboratory, The Francis Crick Institute, London NW1 1AT, UK
[26]UCL Queen Square Institute of Neurology, Queen Square, London WC1N 3BG, UK
[27]Structural Biology of Disease Processes Laboratory, The Francis Crick Institute, London NW1 1AT, UK
[28]RNA Virus Replication Laboratory, The Francis Crick Institute, London NW1 1AT, UK
[29]Retroviral Immunology Laboratory, The Francis Crick Institute, London NW1 1AT, UK
[30]Haemato-oncology Unit, The Royal Marsden NHS Foundation Trust, London SW3 6JJ, UK
[31]Haemato-oncology Unit, The Institute of Cancer Research, London SW7 3RP, UK
[32]Department of Infectious Disease, Imperial College London, London W2 0NN, UK
[33]Melanoma and Kidney Cancer Team, The Institute of Cancer Research, London SW7 3RP, UK
[34]These authors contributed equally
[35]Lead contact
*Correspondence: samra.turajlic@crick.ac.uk
https://doi.org/10.1016/j.xcrm.2022.100781

in patients with solid cancer, but a substantial proportion of patients with blood cancer still lacked NAb responses against Omicron BA.1.[3,4] Additional sublineages with immune-evasive properties, such as BA.4. and BA.5, are now prevalent in many countries, including the UK.[5] Amid widespread lifting of COVID-19 public health measures and high rates of community transmission of Omicron, a significant proportion of COVID-19 deaths still occur in patients with blood cancer.[6] In the UK, a fourth vaccine dose was recommended in December 2021 for patient groups, including patients with blood cancer. It remains unknown whether this has an impact on those with suboptimal responses following three doses.

Here, we report the follow-up findings from CAPTURE, a prospective longitudinal cohort study assessing the functional immune responses to COVID vaccinations in patients with cancer. We report immune responses in patients with blood cancer who received a fourth vaccine dose in December 2021 to February 2022. Longitudinal sampling within CAPTURE was used to identify patients with breakthrough infections (BTIs) and to describe their NAb responses before and after infection.

RESULTS

We evaluated 80 patients with blood cancer who received a third and fourth dose of BNT162b2 after two doses of ChAdOx1 (n = 45, 56%) or BNT162b2 (n = 35, 44%) (Table 1). Furthermore, we evaluated 51 patients (n = 40 solid cancer, n = 11 blood cancer) with BTI at least 7 days following the second or third dose of the COVID-19 vaccine (Table 2).

The breakdown of patients who received a fourth vaccine dose (Table 1) was lymphoma (n = 21), acute leukemia (n = 7), myeloma (n = 33), chronic lymphocytic leukemia (n = 16), and myelodysplastic syndromes (n = 3). Fifteen percent of the patients (n = 12) had confirmed past COVID-19 infection (all prior to the second vaccine dose). Matched post-third- and post-fourth-dose blood samples were available for 76/80 patients. Blood was collected at a median of 28 days (range 8–60 days) after the third dose and 18 days (range 6–67 days) after the fourth dose. NAbs were measured using an established microneutralization assay,[7–9] and IC_{50} titers (NAbT) of <40 (below the quantitative range) were considered undetectable.

Following three vaccine doses, 62% (47/76) of patients with blood cancer had detectable NAbT against Omicron BA.1, compared with 87% (66/76) against wild-type SARS-CoV-2 (Wuhan, hereafter WT) (McNemar test, $p < 0.0001$), and 72% (55/76) against Delta (McNemar test, p = 0.013). Following the fourth vaccine dose, the proportion of patients with detectable NAbT against Omicron BA.1 was 79% (63/80) compared with 98% (78/80) against WT (McNemar test, p = 0.0003) and 78% (62/80) against Delta (McNemar test, p = 1). Significant differences in the proportion of patients with detectable NAbT after three vs. four vaccine doses were apparent for Omicron BA.1 (McNemar test, p = 0.0015) and WT (McNemar test, p = 0.013), but not for Delta (McNemar test, p = 0.51) (Figure 1A).

The BA.1 Omicron sublineage was followed by the several other sublineages (BA.2, BA.4, BA.5). Comparable NAb responses were observed for BA.1 and BA.2 in individuals without cancer.[10] In our cohort, following four vaccine doses, 90% (72/80) of patients with blood cancer had detectable NAbT against BA.2, which was higher than the proportion with NAb against BA.1 (McNemar test, p = 0.008) (Figure 1B).

Multivariable logistic regression analysis (MVA; Table 3) showed that patients treated with B cell-depleting therapies were at a higher risk of having undetectable NAbTs against Omicron BA.1 or BA.2 sublineages after four vaccine doses (anti-CD20 [n = 11] and BTKi [n = 4]; BA.1, OR [95% CI] 0.03 [0.003–0.14], p = 0.0013; BA.2, OR [95% CI] 0.06 [0.004–0.41], p = 0.03). The association of B cell-depleting therapies with low NAbT was confirmed in an ordinal logistic regression model with NAbTs split into three categories (undetectable [≤ 40], moderate [>40–256], and high [>256]) (Tables S1 and S2). Only 3/11 and 7/11 of patients treated with anti-CD20 within

Table 1. Baseline demographic, medical, and oncological history of patients who received a fourth COVID-19 vaccine (n = 80)

	Fourth COVID-19 vaccine dose cohort	AIM T cell assay subset
	n = 80	n = 39
Patient demographics		
Age, years (median, IQR)	63 (55–70)	62 (54–69)
Male, n (%)	47 (59)	24 (62)
Ethnicity, white, n (%)	67 (84)	33 (85)
Vaccination and prior SARS-CoV-2 infection		
First and second COVID-19 vaccine, n (%)		
ChAdOx1	45 (56)	24 (62)
BNT162b2	35 (44)	15 (38)
Third and fourth COVID-19 vaccine, n (%)		
BNT162b2	80 (100)	39 (100)
Time from third to fourth vaccine dose, days (median, IQR)	92 (86–96)	93 (85–97)
Previous SARS-CoV-2 infection, n (%)		
Any time before second vaccine	11 (14)	3 (8)
Cancer and treatment history		
Cancer type, n (%)		
Solid cancer	0 (0)	0 (0)
Blood cancer	80 (100)	39 (100)
Diagnosis, n (%)		
Lymphoma	21 (26)	14 (36)
Myeloma	33 (41)	11 (28)
CLL	16 (20)	12 (31)
Acute leukemia	7 (9)	2 (5)
Myelodysplastic syndrome	3 (4)	0 (0)
Cancer status, n (%)		
Complete response to SACT/remission	37 (46)	16 (41)
Never treated	9 (11)	5 (13)
Progressive disease on SACT/relapse	9 (11)	8 (21)
Partial response to SACT/remission	22 (28)	9 (23)
Stable disease	2 (3)	1 (3)
Rx prior to first vaccine dose, n (%)		
Chemotherapy, <28 days	6 (8)	3 (8)
Targeted therapy, <28 days	25 (31)	8 (21)
Anti-CD20 mAb, <12 months	9 (11)	6 (15)
BTKi therapy, <28 days	4 (5)	3 (8)
No recent SACT	47 (59)	25 (64)
HSCT, any history of	37 (46)	15 (38)
Autograft, any history of	23 (29)	10 (26)
Allograft, any history of	14 (18)	5 (13)

Table 1. ***Continued***

	Fourth COVID-19 vaccine dose cohort	AIM T cell assay subset
	n = 80	n = 39
HSCT, <6 months	4 (5)	1 (3)
CAR-T, <6 months	2 (3)	2 (5)
Rx prior to fourth vaccine dose, n (%)		
Chemotherapy, <28 days	8 (10)	3 (8)
Targeted therapy, <28 days	30 (38)	9 (23)
Anti-CD20 mAb, <12 months	11 (14)	8 (21)
BTKi therapy, <28 days	4 (5)	3 (8)
No recent SACT	38 (48)	24 (62)

AIM T cell assay was performed in a subset of 39 patients. Values are numbers and percentages, n (%), unless otherwise stated. AIM, activation-induced marker; BTKi, Bruton's tyrosine kinase inhibitor; CAR-T, chimeric antigen receptor T cell; CLL, chronic lymphocytic leukemia; HSCT, hematopoietic stem cell transplant; IQR, interquartile range; mAb, monoclonal antibody; Rx, treatment; SACT, systemic anti-cancer therapy.

12 months prior to vaccination and 2/4 and 3/4 patients treated with BTKi within 28 days prior to vaccination had detectable NAbTs against Omicron BA.1 and BA.2, respectively, after four vaccine doses.

$CD4^+$ and $CD8^+$ T cell responses were analyzed using an activation-induced marker (AIM) assay (using CD137 and OX40 as markers for $CD4^+$ T cell activation and CD137 and CD69 as markers for $CD8^+$ T cell activation) after stimulation with a peptide pool against full-length WT spike or Omicron spike in 39/80 patients with blood cancer (Table 1). T cell responses were considered positive if a 2-fold increase in AIM-positive T cells was detected after peptide stimulation vs. unstimulated control.[11] Thirty-two of thirty-nine (82%) patients were evaluable and had matched samples after the third and fourth doses.

Considering T cell responses against Omicron spike, 31% (10/32) of patients with blood cancer had $CD4^+$ T cell responses after three doses compared with 59% (19/32) after four doses (McNemar test, p = 0.0077) (Figure 1C), while 34% (11/32) had $CD8^+$ T cell responses after three and 44% (14/32) after four doses (McNemar test, p = 0.51) (Figure 1D). Considering T cell responses to the WT spike, 59% (19/32) of patients had $CD4^+$ T cell responses after three vaccine doses compared with 81% (26/32) after four doses (McNemar test, p = 0.045), while the proportion of those with $CD8^+$ T cell responses did not change (56% [18/32] after three and four vaccine doses [McNemar test, p = 1]).

Taken together, these data indicate that patients with blood cancer benefit from a fourth vaccine dose, indicated by increases in the proportion of patients with NAb and T cell responses against VOCs.

Within CAPTURE, we identified 57 participants (n = 41 solid cancer, n = 16 blood cancer) with BTI, defined here as a positive SARS-CoV-2 RT-PCR and/or lateral flow antigen test at least 7 days following the second COVID-19 vaccine. All infections were detected during routine clinical care following two vaccine

Table 2. Baseline demographic, clinical, and oncological history for 51 patients with a history of breakthrough infection (defined as a positive SARS-CoV-2 RT-PCR or lateral flow test at least 7 days following the second COVID-19 vaccination)

		Timing of breakthrough infection	
	Breakthrough infection cohort	After second but before third vaccine	After third or fourth vaccine
	n = 57	n = 36	n = 21
Patient demographics			
Age, years (median, IQR)	52 (50–68)	51 (46–68)	63 (56–67)
Male, n (%)	30 (53)	15 (42)	15 (71)
Ethnicity, white, n (%)	49 (86)	30 (83)	19 (90)
COVID-19 vaccination and prior infection			
First and second COVID-19 vaccine, n (%)			
ChAdOx1	33 (58)	25 (69)	8 (38)
BNT162b2	24 (42)	11 (31)	13 (61)
Third COVID-19 vaccine, n (%)			
ChAdOx1	0 (0)	0 (0)	0 (0)
BNT162b2	44 (77)	23 (64)	21 (100)
No third vaccine	13 (23)	13 (36)	–
SARS-CoV-2 infection history, n (%)			
SARS-CoV-2 prior to second vaccination	2 (4)	1 (3)	1 (5)
Breakthrough infection			
Time from last vaccine dose to infection, median (IQR)	79 (66–139)	111 (65–153)	74 (67–88)
Samples available, yes, n (%)			
Between most recent vaccination and infection	26	12	14
Post-infection	50	35	15
WHO severity score, n (%)			
Asymptomatic (WHO score 1)	9 (16)	6 (17)	3 (14)
Mild (WHO score 2–3)	42 (74)	25 (69)	17 (81)
Moderate (WHO score 4–6)	2 (4)	2 (6)	0 (0)
Severe (WHO score 6–10)	4 (7)	3 (8)	1 (5)
Symptoms, n (%)			
Anosmia	13 (23)	10 (28)	3 (14)
Coryza	20 (35)	9 (25)	11 (52)
Cough	29 (51)	17 (48)	12 (57)
Fatigue	16 (28)	9 (25)	7 (33)
Fever	22 (39)	15 (42)	7 (33)
GI symptoms	6 (11)	3 (8)	3 (14)
Headache	6 (11)	3 (8)	3 (14)
Shortness of breath	15 (26)	11 (31)	4 (19)
Medical management for COVID-19, n (%)			
Hospitalization for treatment of COVID-19	6 (11)	5 (14)	1 (5)
Supplemental oxygen therapy	5 (9)	4 (14)	1 (5)
Dexamethasone	5 (9)	4 (11)	1 (5)
IL-6 mAb	3 (5)	3 (8)	0 (0)
Antiviral therapy[a]	8 (14)	3 (8)	5 (24)
Death within 28 days of positive SARS-CoV-2 test, n (%)	4 (5)	3 (8)	1 (5)
Cancer and treatment history			
Cancer diagnosis and stage, n (%)			
Solid cancer stages I–III	13 (23)	12 (33)	1 (5)
Solid cancer stage IV	28 (49)	21 (58)	7 (33)
Blood cancer	16 (28)	3 (8)	13 (62)

(*Continued on next page*)

Table 2. *Continued*

	Breakthrough infection cohort	Timing of breakthrough infection	
		After second but before third vaccine	After third or fourth vaccine
	n = 57	n = 36	n = 21
Cancer status with respect to most recent treatment at time of infection, n (%)			
Complete response to SACT/remission	10 (18)	4 (11)	6 (29)
Progressive disease on SACT/relapse	17 (30)	12 (33)	5 (24)
Partial response to SACT/remission	13 (23)	7 (19)	6 (29)
Stable disease to SACT	6 (11)	4 (11)	2 (10)
Complete resection/NED	11 (19)	9 (25)	2 (10)
Rx prior to SARS-CoV-2 infection, n (%)			
Chemotherapy, <28 days	13 (23)	9 (25)	4 (19)
Targeted therapy, <28 days	17 (30)	10 (28)	7 (33)
Anti-PD-L1 ± anti-CTLA-4, <6 months	7 (12)	7 (19)	0 (0)
Anti-CD20 mAb, <12 months	3 (5)	1 (3)	2 (10)
HSCT, ever	4 (7)	0 (0)	4 (19)
Other medication			
Corticosteroids	4 (7)	4 (11)	0 (0)

Patients are split according to timing of breakthrough infection relative to second or third COVID-19 vaccination. Values are numbers and percentages, n (%), unless otherwise stated. COVID-19, coronavirus disease 2019; CTLA-4, cytotoxic T lymphocyte-associated protein 4; HSCT, hematopoietic stem cell transplant; IL-6, interleukin-6; IQR, interquartile range; mAb, monoclonal antibody; NED, no evidence of disease; Rx, treatment; PD-L1, programmed death ligand-1; SACT, systemic anti-cancer therapy.
[a]Antiviral therapies included sotrovimab (n = 2), remdesivir (n = 4), and molnupiravir (n = 2).

doses (36 patients, n = 33 solid cancer, n = 3 blood cancer) or three or four vaccine doses (21 patients, n = 8 solid cancer, n = 13 blood cancer) (Table 2).

The median time from the most recent vaccine dose to infection was 79 days (IQR 66–139). Most patients had mild COVID-19 (n = 42/57; WHO score 2–3).[12] The most common symptoms were cough (n = 29), fever (n = 22), or coryza (n = 20); and nine patients were asymptomatic (WHO score 1). Six patients had moderate (n = 2, WHO score 4–6) or severe COVID-19 (n = 4, WHO score 7–10) requiring hospitalization and treatment with oxygen therapy (n = 5), corticosteroids (n = 5), and IL-6 monoclonal antibodies (n = 3). Four patients died within 28 days of a positive SARS-CoV-2 test. Eight patients with blood cancer received antiviral therapies or monoclonal antibodies (remdesivir, n = 4; molnupiravir, n = 2; sotrovimab, n = 2) for treatment of acute SARS-CoV-2 infection. Patients with BTI following the second dose were considered as being infected with the Delta variant given the high prevalence of this variant at the time. In contrast, 19/21 patients infected following the third vaccine dose were infected from December 2021 onward at the peak of the Omicron wave, and these infections were subsequently considered as Omicron infections.

Convalescent blood samples were available for 51/57 patients (n = 36 infected after the second dose, and n = 15 infected after the third dose). During convalescence, 32/36 patients with BTI after two doses had detectable NAbT against Delta (Figures 1E and 1F). Following the third dose, 15/15 patients had detectable NAbT against Omicron after infection (Figures 1E and 1G). In addition, blood samples between the most recent vaccine and the infection were available for 25 patients (n = 12 infected after second dose, n = 13 infected after third dose) (Figure 1E). Eight of twelve patients infected after two vaccine doses had undetectable NAbT against Delta or their NAbT declined before infection (Figure 1F), and 8/13 patients infected after three doses had undetectable NAbT against Omicron before infection (Figure 1G). NabTs against WT were detected in all but one patient (after two vaccine doses) before infection.

Notably, patients with Delta and Omicron BTI had evidence of a degree of boosting cross-reactive neutralizing responses against the other variants, consistent with previous reports that cross-reactivity is observed in previously vaccinated patients.[13] Of the three patients with no detectable convalescent NAb, two were blood cancer patients with severe COVID-19 who later died, and one patient had a solid cancer with mild COVID-19. In summary, our data are consistent with published data in healthy individuals[14] in that low variant-specific NAb responses may contribute to infection risk.

DISCUSSION

We demonstrate that patients with blood cancer can benefit from a fourth vaccine dose, even if they had an undetectable response after three doses, especially when considering immune responses to Omicron BA.1 or BA.2. In a cohort of health care workers, a fourth dose of BNT162b2 after three doses of the same vaccine elicited an increase in spike and neutralizing titers,

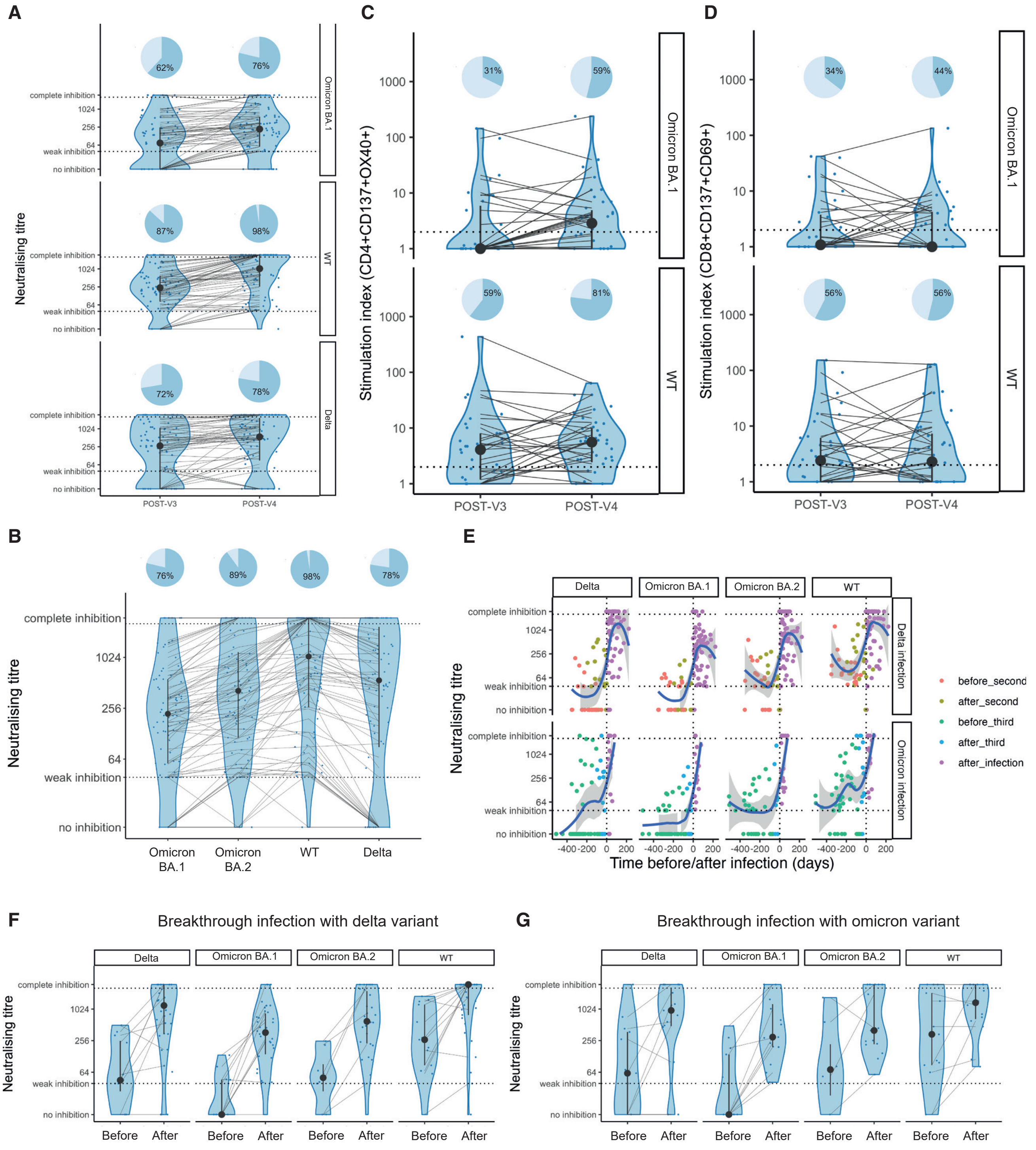

Figure 1. NAb and T cell responses after a fourth vaccine dose and breakthrough infections

(A) NAbTs against Omicron BA.1, WT, and Delta were measured after the fourth vaccine dose. NAbT below (IC_{50} titer <40) or above the quantitative range (IC_{50} titer >2,560) are indicated by horizontal lines.

(B) NAbTs against Omicron BA.1, BA.2, WT, or Delta after four vaccine doses.

(C and D) Levels of (C) $CD4^{+}CD137^{+}OX40^{+}$ or (D) $CD8^{+}CD137^{+}CD69^{+}$ T cells in patients stimulated with WT or Omicron BA.1 full-length spike peptide pools after three or four vaccine doses.

(E) NAbTs against Omicron BA.1, BA.2, WT, and Delta before and after breakthrough infection. Infections after two or three vaccine doses are displayed separately. Timing of blood sampling in relation to vaccination and infection is color-coded. The regression line and 95% CI were fitted using LOESS regression.

(legend continued on next page)

Table 3. Association of clinical parameters with detectable NAb against Omicron

		Detectable NAb against Omicron BA.1		Detectable NAb against Omicron BA.2	
	Patients (n)	OR (95% CI)	p	OR (95% CI)	p
Blood cancer patients	80				
Intercept		2.80 (0.68–15.17)	0.26	3.54 (0.78–23.45)	0.21
Diagnosis (vs. acute leukemia)					
Chronic lymphocytic leukemia	16/80	3.48 (0.46–2.45)	0.30	7.41 (0.78–109.31)	0.16
Myelodysplastic syndrome	3/80	1.21 (0.06–28.03)	0.91	N/A	0.99
Myeloma	33/80	9.53 (0.88–131.13)	0.13	N/A	0.99
Lymphoma	21/80	8.07 (0.88–97.32)	0.13	2.49 (1.90–606.61)	0.06
Vaccine type (first and second dose)					
BNT162b2 (vs. ChAdOx1)	35/80	0.46 (0.13–1.61)	0.31	0.28 (0.04–1.47)	0.23
Previous COVID-19					
SARS-CoV-2 infection before second vaccine dose	11/80	6.52 (1.13–51.90)	0.10	4.08 (0.52–57.99)	0.30
Anti-cancer therapy[a]					
B cell-depleting therapy (anti-CD20 [within 12 months] or BTKi [within 28 days])	15/80	0.03 (0.003–0.14)	0.0013	0.06 (0.004–0.41)	0.04
Chemo- or targeted therapy	34/80	0.68 (0.10–4.17)	0.74	0.55 (0.03–11.24)	0.72

NAbs were binned in detected (≥40) or undetected (<40). All values were caclulated using multivariable binary logistic regression. BTKi, Bruton's tyrosine kinase inhibitor; OR, odds ratio; CI, confidence interval; p, p-value.
[a]For anti-cancer therapy, the indicated treatment was tested for patients who received the treatment vs. patients not receiving that treatment.

surpassing titers immediately after the third dose.[15] Our data show a nuanced picture in patients with blood cancer, where especially NAbTs against Omicron (which were undetectable in a substantial proportion after the third dose) were increased by the fourth dose. These findings highlight the need to consider variant-specific responses in determining which patients may benefit from additional vaccine doses or therapies using antiviral prophylaxis or monoclonal antibodies. Recent reports confirmed a higher risk of Omicron BTIs compared with Delta BTIs in individuals both with and without cancer, likely resulting from Omicron escaping vaccine-induced immunity.[16,17] In keeping with these findings, we observed both Delta and Omicron breakthroughs in our cohort, which were associated with low NAbTs against the respective variant.

Our study has several limitations. First, the heterogeneity and size of the cohort limits subgroup analyses, and specific studies in each cancer type are needed to define risk factors for low NAb responses beyond B cell-depleting therapies and to define the determinants of T cell responses. Second, the precise correlate of protection from BTI remains undefined, and prospective studies are needed to accurately estimate infection risk after three and four vaccine doses in patients with blood cancer. Reports after three vaccine doses confirm the high clinical efficacy of COVID-19 vaccines in the general population and elderly individuals[18–21] and an additional benefit in older and at-risk individuals who had received a fourth dose.[22,23] Comparable data are currently lacking in patients with cancer, but our observations in patients with BTIs agree with models and data in healthy populations suggesting a direct association of NAbT with infection risk,[14,24] although our study was not designed to definitively address a direct association.

Third, we have not generated data on NAbTs against the Omicron subvariants BA.4 and BA.5, which have become the prevalent circulating variants in a number of countries, including the UK.[5] Reports indicate that, while booster vaccination increases responses to all Omicron sublineages, BA.4 and BA.5 show greater immune escape; therefore, patients with blood cancer will likely have less protection against these variants.[25–27]

Overall, our data highlight the benefit of a fourth vaccine dose in patients with blood cancer and confirm that patients with B cell-depleting therapies are at the highest risk of having impaired NAb responses.

Limitations of the study

We acknowledge limitations of our study. First, cohort size and heterogeneity limited subgroup analyses, and larger and/or subtype- or treatment-specific cohorts are needed to evaluate immune responses in particular groups. Second, we were unable to directly assess immune responses to the Omicron sublineages BA.4 and BA.5; these are circulating in many countries and have been reported to show greater degree of immune escape that BA.1 and BA.2 sublineages. Finally, our study did

(F and G) Comparison of NAbTs against Omicron BA.1, BA.2, WT, and Delta before infection (but after last vaccine dose) and after infection in patients with breakthrough infection after (F) two and (G) three vaccine doses. Violin plots denote the density of data, point-range denotes the median and the 25th and 75th percentiles. Patients are indicated as individual data points, and samples from individual patients are connected. The proportions of patients with detectable titers are visualized with pie charts (dark blue denotes patients with IC_{50} titers >40). Biological replicates are patient subjects. There were no technical replicates.

not include proactive monitoring for BTIs, which likely led to their underrepresentation, in particular, asymptomatic infections. While the aim of our study was not to define precise correlates of immune protection, a means to identify patients with suboptimal protection should be a priority for the community. Prospective, adequately powered studies to address this are especially important in view of updated vaccine design and in the context of emergent variants.

STAR★METHODS

Detailed methods are provided in the online version of this paper and include the following:

- KEY RESOURCES TABLE
- RESOURCE AVAILABILITY
  - Lead contact
  - Materials availability
  - Data and code availability
- EXPERIMENTAL MODEL AND SUBJECT DETAILS
  - Study design
  - Study schedule and follow-up
  - Patient data
  - Definition of breakthrough SARS-CoV-2 infection
  - WHO classification of severity of COVID-19
  - Handling of whole blood samples
  - Primary cells: PBMC and plasma isolation from whole blood
  - Serum isolation
  - Cell lines and culture
- METHOD DETAILS
  - Virus variants
  - Virus PCR and sequencing
  - High-throughput live virus micro-neutralisation assay
  - PBMC stimulation assay
  - Activation-induced marker assay
- QUANTIFICATION AND STATISTICAL ANALYSIS
- ADDITIONAL RESOURCES

SUPPLEMENTAL INFORMATION

Supplemental information can be found online at https://doi.org/10.1016/j.xcrm.2022.100781.

ACKNOWLEDGMENTS

The authors have contributed this article on behalf of the CAPTURE Consortium. We thank the clinical leads and subunit teams for recruiting patients to the study, including F. Gronthoud, C. Messiou, D. Cunningham, I. Chau, N. Starling, N. Turner, L. Welsh, R.L. Jones, J. Droney, S. Banerjee, K. Harrington, S. Bhide, A. Okines, A. Reid, and S. Kumar. We thank the CAPTURE trial team, including E. Carlyle, K. Edmonds, and L. Del Rosario, as well as H. Ahmod, L. Holt, M. O'Flaherty, D. Kelly, R. Dhaliwal, N. Ash, M. Mumin, L. Dowdie, K. Kelly, F. Williams, T. Foley, C. Lewis, M. Ndlovu, S. Ali, K. Lingard, S. Sarker, M. Mangwende, N. Hunter, J. Korteweg, A. Murra, N. Shaikh, K. Peat, Mandisa Ndlovu, Kate Cullinan, Fiona Dexter, and Nikhil Rudra. We thank the administrative team that delivered the RMH vaccine program, including E. Mossman and J. Codet-Boise. We thank the following for their assistance with trial conduct and data collection: Laura A. Boos, Nalinie Joharatnam-Hogan, Wanyuan Cui, Javier Pascual, Simon Rodney, Justin Mencel, Olivia Curtis, Clemency Stephenson, Anna Robinson, Bhavna Oza, Sheima Farag, and Isla Leslie. We thank clinical research network nurses for their input with consent and specimen collection, including H. Evans, N. Evans, S. Cooper, S. Jain, S. White, L. Roland, L. Hobbs, and J. Dobbyn. We acknowledge the tremendous support from clinical and research teams at participating units at the Royal Marsden Hospital, including E. Black, A. Dela Rosa, C. Pearce, J. Bazin, L. Conneely, C. Burrows, T. Brown, J. Tai, E. Lidington, H. Hogan, A. Upadhyay, D. Capdeferro, I. Potyka, A. Drescher, F. Baksh, M. Balcorta, C. Da Costa Mendes, J. Amorim, V. Orejudos, and L. Davison.

We thank Prof. Wendy Barclay of Imperial College and the wider Genotype to Phenotype Consortium, for the Alpha and Delta strains used in this study, and Max Whiteley and Thushan I. de Silva at The University of Sheffield and Sheffield Teaching Hospitals NHS Foundation Trust for providing source material. We thank Prof. Gavin Screaton of the University of Oxford for the Omicron strain used in this study. We also thank volunteer staff at the Francis Crick Institute and the Crick COVID-19 Consortium.

Due to the pace at which the field is evolving, we acknowledge researchers of COVID-19, particularly those furthering our understanding of the COVID-19 vaccine-induced immune response. We apologize for the work that was not cited.

This research was funded in part by the National Institute for Health Research (NIHR) Biomedical Research Centre at the Royal Marsden NHS Foundation Trust (RMCC32), Cancer Research UK (CRUK) (grant C50947/A18176). This work was supported by the Francis Crick Institute, which receives its core funding from CRUK (FC001988, FC001218, FC001099, FC001002, FC001078, FC001169, FC001030, FC011104), the UK Medical Research Council (FC001988, FC001218, FC001099, FC001002, FC001078, FC001169, FC001030, FC011104), the Wellcome Trust (FC001988, FC001218, FC001099, FC001002, FC001078, FC001169, FC001030, FC011104), and UK Research and Innovation and the UK Medical Research Council (MR/W005611/1). TRACERx Renal is partly funded by the NIHR Biomedical Research Centre at the Royal Marsden Hospital and the ICR (A109). The CAPTURE study is sponsored by the Royal Marsden NHS Foundation Trust and funded by a Royal Marsden Cancer Charity grant. A.R. is supported by an ESMO clinical research fellowship. R.J.W. and K.A.W. receive support from Rosetrees (M926). A.F. has received funding from the European Union's Horizon 2020 research and innovation program under Marie Skłodowska-Curie grant agreement 892360. S.T.C.S. is supported and funded by a CRUK Clinician PhD Fellowship award. L.A. is funded by the Royal Marsden Cancer Charity. F.B. is funded by Rosetrees Charity (grant reference M829). S.T. is funded by CRUK (grant reference C50947/A18176), the NIHR Biomedical Research Centre at the Royal Marsden Hospital and the Institute of Cancer Research (grant reference A109), the Kidney and Melanoma Cancer Fund of the Royal Marsden Cancer Charity, the Rosetrees Trust (grant reference A2204), Ventana Medical Systems (grant reference 10467 and 10530), the National Institutes of Health (USA), and the Melanoma Research Alliance. A.M.S. received an educational grant from Janssen-Cilag. C.S. is funded by CRUK (TRACERx, PEACE, and CRUK Cancer Immunotherapy Catalyst Network), the CRUK Lung Cancer Centre of Excellence (C11496/A30025), the Rosetrees Trust, Butterfield and Stoneygate Trusts, the Novo Nordisk Foundation (ID16584), a Royal Society Professorship Enhancement award (RP/EA/180007), the NIHR Biomedical Research Centre at University College London Hospitals, the CRUK University College London Centre, the Experimental Cancer Medicine Centre, and the Breast Cancer Research Foundation (BCRF 20-157). This work was supported by a Stand Up To Cancer-LUNGevity-American Lung Association Lung Cancer Interception Dream Team Translational research grant (SU2C-AACR-DT23-17 to S.M.D. and A.E.S.). Stand Up To Cancer is a division of the Entertainment Industry Foundation. Research grants are administered by the American Association for Cancer Research, the scientific partner of SU2C. C.S. received an ERC Advanced Grant (PROTEUS) from the European Research Council under the European Union's Horizon 2020 research and innovation program (grant agreement 835297). C.S. is a Royal Society Napier Research Professor (RP150154). R.J.W. has received funding from the Francis Crick Institute, which receives its core funding by Wellcome (FC0010218), UKRI (FC0010218), and CRUK (FC0010218) and research funding from Wellcome (203135 and 222754), Rosetrees (M926), and the South African MRC. The graphical abstract was created in BioRender.com.

CellPress
OPEN ACCESS

AUTHOR CONTRIBUTIONS

Conceptualization, A.F., S.T.C.S., L.A., and S.T.; data curation, A.F., S.T.C.S., L.A., M.W., R.H., A.M.S., Z.T., B.S., S.F., A.R., T.F., E.C., and K.E.; formal analysis, A.F., S.T.C.S., L.A., and S.T.; funding acquisition, A.F., L.A., and S.T.; investigation, A.F., S.T.C.S., L.A., M.W., R.H., K.W., T.B., A.E.-H., N.C.-L., F.B., D.D., S.K., O.-R.S., C.Q., C.K., K.P., D.K., A.M., K.K., M.O'F., N.Y., S.J., D.C., N.V.A., K.Y., A.J.S.F., L.P., E.N., and S.T.; methodology, A.F., M.W., R.H., and K.W.; project administration, A.F., S.T.C.S., L.A., E.C., K.E., L.D.R., K.L., M.M., L.H., H.A., J.K., and T.F.; project oversight and governance, S.T.; resources, M.W., R.H., E.C.W., E.J.C., S.C., J.I.M., R.L.S., G.G., D.M., S.G., S.G., D.L.V.B., K.C.T., S.W., J.L., and S.T.; software, M.G.; supervision, G.K., R.B., C.S., G.K., M.H., E.N., S.W., R.W., J.L., and S.T.; validation, A.F., S.T.C.S., L.A., E.C.W., E.J.C., D.L.V.B., and M.W.; data verification, A.F. and S.T.C.S.; visualization, A.F., S.T.C.S., L.A., and S.T.; writing – original draft, A.F., S.T.C.S., L.A., and S.T.; writing – review & editing, all authors; decision to submit manuscript, S.T.

DECLARATION OF INTERESTS

The authors declare no competing interest.

INCLUSION AND DIVERSITY

We support inclusive, diverse, and equitable conduct of research.

Received: June 6, 2022
Revised: September 7, 2022
Accepted: September 21, 2022
Published: October 18, 2022

REFERENCES

1. Fendler, A., Shepherd, S.T.C., Au, L., Wilkinson, K.A., Wu, M., Schmitt, A.M., Tippu, Z., Farag, S., Rogiers, A., Harvey, R., et al. (2022). Immune responses following third COVID-19 vaccination are reduced in patients with hematologic malignancies compared to patients with solid cancer. Cancer Cell *40*, 114–116. https://doi.org/10.1016/j.ccell.2021.12.013.
2. Shapiro, L.C., Thakkar, A., Campbell, S.T., Forest, S.K., Pradhan, K., Gonzalez-Lugo, J.D., Quinn, R., Bhagat, T.D., Choudhary, G.S., McCort, M., et al. (2022). Efficacy of booster doses in augmenting waning immune responses to COVID-19 vaccine in patients with cancer. Cancer Cell *40*, 3–5. https://doi.org/10.1016/j.ccell.2021.11.006.
3. Aleman, A., Van Oekelen, O., Upadhyaya, B., Beach, K., Kogan Zajdman, A., Alshammary, H., Serebryakova, K., Agte, S., Kappes, K., Gleason, C.R., et al. (2022). Augmentation of humoral and cellular immune responses after third-dose SARS-CoV-2 vaccination and viral neutralization in myeloma patients. Cancer Cell *40*, 441–443. https://doi.org/10.1016/j.ccell.2022.03.013.
4. Fendler, A., Shepherd, S.T.C., Au, L., Wu, M., Harvey, R., Schmitt, A.M., Tippu, Z., Shum, B., Farag, S., Rogiers, A., et al. (2022). Omicron neutralising antibodies after third COVID-19 vaccine dose in patients with cancer. Lancet *399*, 905–907. https://doi.org/10.1016/S0140-6736(22)00147-7.
5. Xia, S., Wang, L., Zhu, Y., Lu, L., and Jiang, S. (2022). Origin, virological features, immune evasion and intervention of SARS-CoV-2 Omicron sublineages. Signal Transduct. Targeted Ther. *7*, 241. https://doi.org/10.1038/s41392-022-01105-9.
6. ONS. https://www.ons.gov.uk/peoplepopulationandcommunity/birthsdeathsandmarriages/deaths/datasets/preexistingconditionsofpeoplewhodiedduetocovid19englandandwales. https://www.ons.gov.uk/peoplepopulationandcommunity/birthsdeathsandmarriages/deaths/datasets/preexistingconditionsofpeoplewhodiedduetocovid19englandandwales.
7. Wall, E.C., Wu, M., Harvey, R., Kelly, G., Warchal, S., Sawyer, C., Daniels, R., Adams, L., Hobson, P., Hatipoglu, E., et al. (2021). AZD1222-induced neutralising antibody activity against SARS-CoV-2 Delta VOC. Lancet *398*, 207–209. https://doi.org/10.1016/S0140-6736(21)01462-8.
8. Wall, E.C., Wu, M., Harvey, R., Kelly, G., Warchal, S., Sawyer, C., Daniels, R., Hobson, P., Hatipoglu, E., Ngai, Y., et al. (2021). Neutralising antibody activity against SARS-CoV-2 VOCs B.1.617.2 and B.1.351 by BNT162b2 vaccination. Lancet *397*, 2331–2333. https://doi.org/10.1016/S0140-6736(21)01290-3.
9. Wu, M., Wall, E.C., Carr, E.J., Harvey, R., Townsley, H., Mears, H.V., Adams, L., Kjaer, S., Kelly, G., Warchal, S., et al. (2022). Three-dose vaccination elicits neutralising antibodies against omicron. Lancet *399*, 715–717. https://doi.org/10.1016/S0140-6736(22)00092-7.
10. Yamasoba, D., Kimura, I., Nasser, H., Morioka, Y., Nao, N., Ito, J., et al. (2022). Virological characteristics of the SARS-CoV-2 Omicron BA.2 spike. Cell *185*, 2103–2115.e2119.
11. Fendler, A., Au, L., Shepherd, S.T.C., Byrne, F., Cerrone, M., Boos, L.A., Rzeniewicz, K., Gordon, W., Shum, B., Gerard, C.L., et al. (2021). Functional antibody and T cell immunity following SARS-CoV-2 infection, including by variants of concern, in patients with cancer: the CAPTURE study. Nat. Can. *2*, 1321–1337. https://doi.org/10.1038/s43018-021-00275-9.
12. Marshall, J.C., Murthy, S., Diaz, J., Adhikari, N.K., Angus, D.C., Arabi, Y.M., Baillie, K., Bauer, M., Berry, S., Blackwood, B., et al. (2020). A minimal common outcome measure set for COVID-19 clinical research. Lancet Infect. Dis. *20*, e192–e197. https://doi.org/10.1016/S1473-3099(20)30483-7.
13. Richardson, S.I., Madzorera, V.S., Spencer, H., Manamela, N.P., van der Mescht, M., Lambson, B.E., et al. (2022). SARS-CoV-2 Omicron triggers cross-reactive neutralization and Fc effector functions in previously vaccinated, but not unvaccinated individuals. Cell Host & Microbe *30* (6), 880–886.e4, In press. https://doi.org/10.1016/j.chom.2022.03.029.
14. Bergwerk, M., Gonen, T., Lustig, Y., Amit, S., Lipsitch, M., Cohen, C., Mandelboim, M., Levin, E.G., Rubin, C., Indenbaum, V., et al. (2021). Covid-19 breakthrough infections in vaccinated health care workers. N. Engl. J. Med. *385*, 1474–1484. https://doi.org/10.1056/NEJMoa2109072.
15. Regev-Yochay, G., Gonen, T., Gilboa, M., Mandelboim, M., Indenbaum, V., Amit, S., Meltzer, L., Asraf, K., Cohen, C., Fluss, R., et al. (2022). Efficacy of a fourth dose of covid-19 mRNA vaccine against omicron. N. Engl. J. Med. *386*, 1377–1380. https://doi.org/10.1056/NEJMc2202542.
16. Mair, M.J., Mitterer, M., Gattinger, P., Berger, J.M., Trutschnig, W., Bathke, A.C., Gansterer, M., Berghoff, A.S., Laengle, S., Gottmann, L., et al. (2022). Enhanced SARS-CoV-2 breakthrough infections in patients with hematologic and solid cancers due to Omicron. Cancer Cell *40*, 444–446. https://doi.org/10.1016/j.ccell.2022.04.003.
17. Chaguza, C., Coppi, A., Earnest, R., Ferguson, D., Kerantzas, N., Warner, F., Young, H.P., Breban, M.I., Billig, K., Koch, R.T., et al. (2022). Rapid emergence of SARS-CoV-2 Omicron variant is associated with an infection advantage over Delta in vaccinated persons. Med *3*, 325–334.e4. https://doi.org/10.1016/j.medj.2022.03.010.
18. Abu-Raddad, L.J., Chemaitelly, H., Ayoub, H.H., AlMukdad, S., Yassine, H.M., Al-Khatib, H.A., Smatti, M.K., Tang, P., Hasan, M.R., Coyle, P., et al. (2022). Effect of mRNA vaccine boosters against SARS-CoV-2 omicron infection in Qatar. N. Engl. J. Med. *386*, 1804–1816. https://doi.org/10.1056/NEJMoa2200797.
19. Andrews, N., Stowe, J., Kirsebom, F., Toffa, S., Rickeard, T., Gallagher, E., Gower, C., Kall, M., Groves, N., O'Connell, A.M., et al. (2022). Covid-19 vaccine effectiveness against the omicron (B.1.1.529) variant. N. Engl. J. Med. *386*, 1532–1546. https://doi.org/10.1056/NEJMoa2119451.
20. Baum, U., Poukka, E., Leino, T., Kilpi, T., Nohynek, H., and Palmu, A.A. (2022). High vaccine effectiveness against severe Covid-19 in the elderly in Finland before and after the emergence of Omicron. Preprint at medRxiv. https://doi.org/10.1101/2022.03.11.22272140.
21. Lauring, A.S., Tenforde, M.W., Chappell, J.D., Gaglani, M., Ginde, A.A., McNeal, T., Ghamande, S., Douin, D.J., Talbot, H.K., Casey, J.D., et al. (2022). Clinical severity of, and effectiveness of mRNA vaccines against,

covid-19 from omicron, delta, and alpha SARS-CoV-2 variants in the United States: prospective observational study. BMJ *376*, e069761. https://doi.org/10.1136/bmj-2021-069761.

22. Bar-On, Y.M., Goldberg, Y., Mandel, M., Bar-On, Y.M., Bodenheimer, O., Freedman, L., et al. (2022). Protection by 4th dose of BNT162b2 against omicron in Israel. New England Journal of Medicine *386* (*18*), 1712–1720, In press. https://doi.org/10.1056/NEJMoa2201570.
23. Ronen, A., Ruslan, S., Michael, F., Peretz, A., Beckenstein, T., Yaron, S., et al. (2022). Second booster vaccine and Covid-19 mortality in adults 60 to 100 years old. Nature Medicine *28* (*7*), 1486–1490, In press. https://doi.org/10.1038/s41591-022-01832-0.
24. Khoury, D.S., Cromer, D., Reynaldi, A., Schlub, T.E., Wheatley, A.K., Juno, J.A., Subbarao, K., Kent, S.J., Triccas, J.A., and Davenport, M.P. (2021). Neutralizing antibody levels are highly predictive of immune protection from symptomatic SARS-CoV-2 infection. Nat. Med. *27*, 1205–1211. https://doi.org/10.1038/s41591-021-01377-8.
25. Yao, L., Zhu, K.-L., Jiang, X.-L., Wang, X.J., Zhan, B.D., Gao, H.X., Geng, X.Y., Duan, L.J., Dai, E.H., and Ma, M.J. (2022). Omicron subvariants escape antibodies elicited by vaccination and BA.2.2 infection. Lancet Infect. Dis. *22*, 1116–1117. https://doi.org/10.1016/S1473-3099(22)00410-8.
26. Bowen, J.E., Addetia, A., Dang, H.V., Stewart, C., Brown, J.T., Sharkey, W.K., Sprouse, K.R., Walls, A.C., Mazzitelli, I.G., Logue, J.K., et al. (2022). Omicron spike function and neutralizing activity elicited by a comprehensive panel of vaccines. Science *377*, 890–894. https://doi.org/10.1126/science.abq0203.
27. Cao, Y., Wang, J., Jian, F., Xiao, T., Song, W., Yisimayi, A., Huang, W., Li, Q., Wang, P., An, R., et al. (2022). Omicron escapes the majority of existing SARS-CoV-2 neutralizing antibodies. Nature *602*, 657–663. https://doi.org/10.1038/s41586-021-04385-3.
28. Faulkner, N., Ng, K.W., Wu, M.Y., Harvey, R., Margaritis, M., Paraskevopoulou, S., Houlihan, C., Hussain, S., Greco, M., Bolland, W., et al. (2021). Reduced antibody cross-reactivity following infection with B.1.1.7 than with parental SARS-CoV-2 strains. Elife *10*, e69317. https://doi.org/10.7554/eLife.69317.
29. Au, L., Boos, L.A., Swerdlow, A., Byrne, F., Shepherd, S.T.C., Fendler, A., and Turajlic, S.; CAPTURE investigators (2020). Cancer, COVID-19, and antiviral immunity: the CAPTURE study. Cell *183*, 4–10. https://doi.org/10.1016/j.cell.2020.09.005.
30. Fendler, A., Shepherd, S.T.C., Au, L., Wilkinson, K.A., Wu, M., Byrne, F., Cerrone, M., Schmitt, A.M., Joharatnam-Hogan, N., Shum, B., et al. (2021). Adaptive immunity and neutralizing antibodies against SARS-CoV-2 variants of concern following vaccination in patients with cancer: the CAPTURE study *2*, 1305–1320. https://doi.org/10.1038/s43018-021-00274-w.
31. Fessas, P., Lee, H., Ikemizu, S., and Janowitz, T. (2017). A molecular and preclinical comparison of the PD-1-targeted T-cell checkpoint inhibitors nivolumab and pembrolizumab. Semin. Oncol. *44*, 136–140. https://doi.org/10.1053/j.seminoncol.2017.06.002.
32. van den Brink, E.N., ter Meulen, J., Cox, F., Jongeneelen, M.A.C., Thijsse, A., Throsby, M., Marissen, W.E., Rood, P.M.L., Bakker, A.B.H., Gelderblom, H.R., et al. (2005). Molecular and biological characterization of human monoclonal antibodies binding to the spike and nucleocapsid proteins of severe acute respiratory syndrome coronavirus. J. Virol. *79*, 1635–1644. https://doi.org/10.1128/JVI.79.3.1635-1644.2005.

STAR★METHODS

KEY RESOURCES TABLE

REAGENT or RESOURCE	SOURCE	IDENTIFIER
Antibodies		
V500 Mouse Anti-Human CD14	BD	Cat#561391; RRID:AB_10611856
V500 Mouse anti-Human CD19	BD	Cat#561121; RRID:AB_1056239
Brilliant Violet 605 anti-human CD4 Antibody	Biolegend	Cat#317438
Brilliant Violet 650^{TM} anti-human CD8a Antibody	Biolegend	Cat#301042
PE-CF594 Mouse Anti-Human CD69	BD	Cat#562617; RRID:AB_2737680
PE/Cyanine7 anti-human CD134 (OX40) Antibody	Biolegend	Cat#350012
APC anti-human CD137 (4-1BB) Antibody	Biolegend	Cat#309810; RRID:AB_830672
Alexa Fluor® 700 anti-human CD3 Antibody	Biolegend	Cat#317340; RRID:AB_2563408
Alexa488-labelled-CR3009 anti-SARS-CoV-2 Ab	produced in-house	
CD3022 anti-SARS-CoV-2 Ab	Absolute Antibodies	Cat#Ab01680-10.0
Bacterial and virus strains		
MS066352H - B.1.617.2 (“Delta”) isolate	Prof. Wendy Barclay, Imperial College London, London, UK	GISAID accession number: EPI_ISL_1731019
M21021166 - BA.1 (“Omicron”) isolate	Prof. Gavin Screaton, University of Oxford, Oxford, UK	
Crick179 - BA.2 isolate	Emma Wall, Mary Wu, The Francis Crick Institute, London, UK	
hCoV19/England/02/2020 - "WT" SARS-CoV-2 isolate	Respiratory Virus Unit, Public Health England	GISAID accession number: EPI_ISL_407073
MS066352H - B.1.617.2 (“Delta”) isolate	Prof. Wendy Barclay, Imperial College London, London, UK	GISAID accession number: EPI_ISL_1731019
M21021166 - BA.1 (“Omicron”) isolate	Prof. Gavin Screaton, University of Oxford, Oxford, UK	
Crick179 - BA.2 isolate	Emma Wall, Mary Wu, The Francis Crick Institute, London, UK	
hCoV19/England/02/2020 - "WT" SARS-CoV-2 isolate	Respiratory Virus Unit, Public Health England	GISAID accession number: EPI_ISL_407073
Experimental models: Cell lines		
VERO-E6	Dr Björn Meyer, Institut Pasteur, Paris, France[28]	
Peripheral blood mononuclear cells (primary cells)	CAPTURE study participants	NCT03226886
Chemicals, peptides, and recombinant proteins		
Custom omicron BA.1 peptide pool (15-mer sequences with 11 amino acids overlap covering the complete S-protein)	Pepscan	Cat#8907693
PepTivator SARS-CoV-2 spike (S) (Miltenyi Biotec) (synthetic SARS-CoV-2 peptide pools, consisting of 15-mer sequences with 11 amino acid overlap covering the complete S protein)	Miltenyi Biotec	Cat#130-126-700

RESOURCE AVAILABILITY

Lead contact

Further information and requests for resources and reagents should be directed to and will be fulfilled by the lead contact, Samra Turajlic (samra.turajlic@crick.ac.uk).

Materials availability

All requests for resources and reagents should be directed to the lead contact author. All reagents will be made available on request after completion of a Materials Transfer Agreement.

Data and code availability

- Data: All data reported in this paper will be shared by the lead contact upon request.
- Code: This paper does not report original code.
- Additional information: Any additional information required to reanalyze the data reported in this paper is available from the lead contact upon request.

EXPERIMENTAL MODEL AND SUBJECT DETAILS

Study design

CAPTURE (NCT03226886) is a prospective, longitudinal cohort study that commenced recruitment in May 2020 at the Royal Marsden NHS Foundation Trust. Adult patients with a current diagnosis or history of invasive cancer are eligible for enrolment.[29] Inclusion criteria are intentionally broad, and patients were recruited irrespective of cancer type, stage, or treatment. The primary endpoint of the CAPTURE study was the seroconversion rate in cancer patients at 14–28 days following the second dose of vaccine.[30] Exploratory endpoints include evaluation of neutralising responses to SARS-CoV-2 variants of concern (VOC).

CAPTURE received ethical approval as a substudy of the TRACERx Renal Study (NCT03226886). TRACERx Renal was initially approved by the NRES Committee London, Fulham, on January 17, 2012 (11/LO/1996). The CAPTURE protocol was part of Substantial Amendment 9 and received approval by the Health Research Authority on April 30, 2020, and the NRES Committee London, Fulham on May 1, 2020. CAPTURE is conducted in accordance with the ethical principles of the Declaration of Helsinki, Good Clinical Practice and applicable regulatory requirements. All patients provided written, informed consent to participate. The Chief Investigator, Samra Turajlic is responsible for the oversight of all aspects of study conduct and governance.

Study schedule and follow-up

Detailed sampling schedule and methodology were described previously.[29] Patients eligible for a third and fourth vaccine dose were invited to receive the vaccine in our institution. Samples were collected following the third vaccine dose (Post-V3; 14–28 days post third vaccination) and following fourth vaccine dose (Post-V4; 7–28 days post fourth vaccine dose).

The study protocol did not mandate screening for breakthrough SARS-CoV-2 infections and all breakthrough infections were detected during the course of routine clinical care. Where breakthrough infections were reported, an additional post-infection blood sample was sought at least 14 days following the positive SARS-CoV-2 test.

Patient data

Demographic, epidemiological and clinical data (e.g. cancer type, cancer stage, treatment history, history of SARS-CoV-2 infection) were collected from the internal electronic patient record and prospectively from patients. Pseudonymised data was entered into a cloud-based electronic database (Ninox Software, Berlin, Germany). Chemotherapy, targeted therapy (small molecule inhibitors or monoclonal antibodies) or endocrine therapy was deemed to be current if given within 28 days of vaccination. Treatment with immune checkpoint inhibitors (CPI) within six months was considered significant given the prolonged receptor occupancy reported with these agents.[31] Treatment with ant-CD20 monoclonal antibodies within 12 months was considered. Concomitant medications were recorded for: corticosteroids (considered significant if >10 mg prednisolone equivalent given for at least seven days); GCSF when delivered within 48 h of vaccination or five days in the case of pegylated preparation; and other immunosuppressive drugs taken within 48 h of vaccination.

Definition of breakthrough SARS-CoV-2 infection

We considered patients to have had a breakthrough SARS-CoV-2 infection if they had SARS-CoV-2 positive RT-PCR (tests conducted as part of routine clinical care) at least seven days following the second COVID-19 vaccine dose. Breakthrough infections after the second vaccine dose were considered delta infections while breakthrough infections after the third vaccine dose were considered omicron infections based on the high prevalence of the respective variants at the time.

WHO classification of severity of COVID-19

We classified the severity of COVID-19 according to the WHO ordinal clinical progression scale.[12] Uninfected: uninfected, no viral RNA detected – 0; Asymptomatic: viral RNA and/or S1-reactive IgG detected – 1; mild (ambulatory): symptomatic, independent – 2; symptomatic, assistance needed - 3; moderate (hospitalised): no oxygen therapy (if hospitalised for isolation only, record status as for ambulatory patient) – 4; oxygen by mask or nasal prongs - 5; severe (hospitalised): oxygen by non-invasive ventilation or high

flow – 6; intubation and mechanical ventilation, $pO_2/FiO_2 \geq 150$ or $SpO_2/FiO_2 \geq 200$ – 7; mechanical ventilation, $pO_2/FiO_2 < 150$ ($SpO_2/FiO_2 < 200$) or vasopressors – 8; mechanical ventilation, $pO_2/FiO_2 < 150$ and vasopressors, dialysis, or extracorporeal membrane oxygenation - 9; Dead - 10.

Handling of whole blood samples

All blood samples and isolated products were handled in a CL2 laboratory inside a biosafety cabinet using appropriate personal protective equipment and safety measures, in accordance with a risk assessment and standard operating procedure approved by the safety, health and sustainability committee of the Francis Crick Institute.

Primary cells: PBMC and plasma isolation from whole blood

All primary cells in this study were procured from CAPTURE participants. Whole blood was collected in EDTA tubes (VWR) and stored at 4°C until processing. All samples were processed within 24 h. Time of blood draw, processing, and freezing was recorded. Prior to processing, tubes were brought to room temperature (RT). PBMC and plasma were isolated by density-gradient centrifugation using pre-filled centrifugation tubes (pluriSelect). Up to 30 mL of undiluted blood was added on top of the sponge and centrifuged for 30 min at 1000 g at RT. Plasma was carefully removed then centrifuged for 10 min at 4000 g to remove debris, aliquoted and stored at −80°C. The cell layer was then collected and washed twice in PBS by centrifugation for 10 min at 300 g at RT. PBMC were resuspended in Recovery cell culture freezing medium (Fisher Scientific) containing 10% DMSO, placed overnight in freezing containers (Corning) at −80°C and then transferred for long-term storage in liquid nitrogen. PBMCs for *in vitro* stimulation were thawed at 37°C and resuspended in 10 mL of warm complete medium (RPMI and 5% human AB serum) containing 0.02% benzonase. 2×10^6 cells were seeded in 200 μL complete medium in 96-well plates and cultured for 24 h at 37°C, 5% CO2.

Serum isolation

Whole blood was collected in serum coagulation tubes (Vacuette CAT tubes, Greiner) for serum isolation and stored at 4°C until processing. All samples were processed within 24 h. Time of blood draw, processing, and freezing was recorded. Tubes were centrifuged for 10 min at 2000 g at 4°C. Serum was separated from the clotted portion, aliquoted and stored at −80°C.

Cell lines and culture

Vero E6 cells were kindly provided by Dr Björn Meyer, Institut Pasteur, Paris, France. Cells were grown in Iscove's Modified Dulbecco's Medium (Sigma-Aldrich) supplemented with 5% fetal bovine serum (Thermo Fisher Scientific), L-glutamine (2 mM, Thermo Fisher Scientific), penicillin (100 U/mL, Thermo Fisher Scientific), and streptomycin (0.1 mg/mL, Thermo Fisher Scientific).

METHOD DETAILS

Virus variants

The SARS-CoV-2 reference isolate (referred to as 'WT') was hCoV19/England/02/2020, obtained from the Respiratory Virus Unit, Public Health England (GISAID EpiCov accession, EPI_ISL_407073). The B.1.617.2 ("Delta") isolate was MS066352H (GISAID accession number EPI_ISL_1731019), which carries the T19R, K77R, G142D, Δ156-157/R158G, A222V, L452R, T478K, D614G, P681R, D950N, and was kindly provided by Prof. Wendy Barclay, Imperial College London, London, UK through the Genotype-to-Phenotype National Virology Consortium (G2P-UK). The BA.1 ("Omicron") isolate was M21021166, which carries the A67V, Δ69–70, T95I, Δ142-144, Y145D, Δ211, L212I, G339D, S371L, S373P, S375F, K417N, N440K, G446S, S477N, T478K, E484A, Q493R, G496S, Q498R, N501Y, Y505H, T547K, D614G, H655Y, N679K, P681H, A701V, N764K, D796Y, N856K, Q954H, N969K, and L981F mutations in Spike. It was kindly provided by Prof. Gavin Screaton, University of Oxford, Oxford, UK through the Genotype-to-Phenotype National Virology Consortium (G2P-UK). The BA.2 isolate was Crick179, isolated from a nasopharyngeal swab collected from a participant in the UCLH-Crick Legacy study.[7–9] Swabs were collected in Vital-Transport medium (VTM), transported, and stored at 4° prior to viral culture. This isolate carries the T19I, L24_A27del, G142D, V213G, G339D, S371F, S373P, S375F, T376A, D405N, R408S, K417N, N440K, S477N, T478K, E484A, Q493R, Q498R, N501Y, Y505H, D614G, H655Y, N679K, P681H, N764K, D796Y, Q954H mutations in Spike. All viral isolates were propagated in Vero E6 cells. Briefly, 50% confluent monolayers of Vero E6 cells were infected with the given SARS CoV-2 strains at an MOI of approx. 0.001. Cells were washed once with DMEM (Sigma; D6429), then 5 mL virus inoculum made up in DMEM was added to each T175 flask and incubated at room temperature for 30 min. DMEM + 1% FCS (Biosera; FB-1001/500) was added to each flask. Cells were incubated at 37°C, 5% CO^2 for four days until the extensive cytopathogenic effect was observed. The supernatant was harvested and clarified by centrifugation at 2000 rpm for 10 min in a benchtop centrifuge. The supernatant was aliquoted and frozen at −80°C.

Virus PCR and sequencing

All virus stocks generated for use in neutralisation assays were sequence-validated before use. To confirm the identity of cultured VoC samples, 8ul of viral RNA was prepared for sequencing by the ARTIC method (https://www.protocols.io/view/ncov-2019-sequencingprotocol-v3-locost-bh42j8ye) and sequenced on the ONT GridION platform to >30k reads/sample. The data was demultiplexed and processed using the viralrecon pipeline (https://github.com/nf-core/viralrecon).

High-throughput live virus micro-neutralisation assay

High-throughput live virus micro-neutralisation assays were performed as described previously.[28] Briefly, Vero E6 cells (Institute Pasteur) at 90–100% confluency in 384-well format were first titrated with varying MOI of each SARS-CoV-2 variant and varying concentrations of a control monoclonal nanobody to normalise for possible replicative differences between variants and select conditions equivalent to wild-type virus. Following this calibration, cells were infected in the presence of serial dilutions of patient serum samples. After infection (24 h), cells were fixed with 4% final Formaldehyde, permeabilised with 0.2% TritonX-100, 3% BSA in PBS (v/v), and stained for SARS-CoV-2 N protein using Alexa488-labelled-CR3009 antibody produced in-house and cellular DNA using DAPI.[32] Whole-well imaging at 5× was carried out using an Opera Phenix (Perkin Elmer) and fluorescent areas and intensity calculated using the Phenix-associated software Harmony 9 (Perkin Elmer). Inhibition was estimated from the measured area of infected cells/total area occupied by all cells. The inhibitory profile of each serum sample was estimated by fitting a 4-parameter dose-response curve executed in SciPy. Neutralising antibody titres are reported as the fold-dilution of serum required to inhibit 50% of viral replication (IC_{50}). They are further annotated if they lie above the quantitative (complete inhibition) range, below the quantitative range but still within the qualitative range (i.e. partial inhibition is observed, but a dose-response curve cannot be fit because it does not sufficiently span the IC_{50}), or if they show no inhibition at all. IC_{50} values above the quantitative limit of detection of the assay (>2560) were recoded as 3000; IC_{50} values below the quantitative limit of the assay (<40) but within the qualitative range were recoded as 39 and data below the qualitative range (i.e. no response observed) were recoded as 10.

PBMC stimulation assay

PBMCs for *in vitro* stimulation were thawed at 37°C and resuspended in 10 mL of warm complete medium (RPMI and 5% human AB serum) containing 0.02% benzonase. 2×10^6 cells were seeded in 200 μL complete medium in 96-well plates. Cells were stimulated with 4 μL per well PepTivator SARS-CoV-2 spike (S) (Miltenyi Biotec) (synthetic SARS-CoV-2 peptide pools, consisting of 15-mer sequences with 11 amino acid overlap covering the complete S protein), or a custom Omicron BA.1 spike peptide pool (Pepscan) (15-mer sequences with 11 amino acids overlap covering the complete S-protein) representing 1 μg mL−1 final concentration per peptide. SEB (Merck, UK) was used as a positive control at 0.5 μg mL−1 final concentration, negative control was PBS containing dimethylsulfoxide at 0.002% final concentration. PBMCs were cultured for 24 h at 37°C, 5% CO2.

Activation-induced marker assay

Cells were washed twice in warm PBMC medium. Dead cells were stained with 0.5 μL per well Zombie dye V500 for 15 min at room temperature in the dark, then washed once with PBS containing 2% FCS (FACS buffer). A surface staining mix was prepared, containing 1 μL per well of each antibody in 50:50 brilliant stain buffer (BD) and FACS buffer. PBMCs were stained with 50 μL surface staining mix for 30 min at room temperature in the dark. Cells were washed once in FACS buffer and fixed in 1% PFA in FACS buffer for 20 min, then washed once and resuspended in 200 μL PBS. All samples were acquired on a Bio-Rad Ze5 flow cytometer running Bio-Rad Everest software v.2.4 and analyzed using FlowJo v.10.7.1 (Tree Star). Compensation was performed with 20 μL antibody-stained anti-mouse Ig, κ/negative control compensation particle set (BD Biosciences). A total of 1×10^6 live $CD3^+CD19^-CD14^-$ cells were acquired per sample. Gates were drawn relative to the unstimulated control for each donor. CD137 and OX40 were used to quantify $CD4^+$ T cell activation, CD137 and CD69 were used for $CD8^+$ T cell activation. T cell response are reported as a stimulation index by dividing the percentage of activation-induced marker (AIM)-positive cells by the percentage of cells in the negative control. If negative control was 0, then the minimum value across the cohort was used. A 2-fold increase in stimulation index was considered positive.

QUANTIFICATION AND STATISTICAL ANALYSIS

Data and statistical analysis were done in R v3.6.1 in R studio v1.2.1335. McNemar and Wilcoxon Mann-Whitney-U test were used to evaluate statistical significance. A p value <0.05 was considered significant. All tests were performed two-sided. Statistical details for each experiment are provided in the figure legends. The ggplot2 package in R was used for data visualisation. Data are usually plotted as single data points and violin plots on a logarithmic scale. PointRange in violin plots denotes median and upper and lower quartiles. For breakthrough infection trends in NAbT are visualised with a loess regression curve. Multivariable binary logistic regression analysis was performed using the glm function within the stats package in R, OR and 95% CI were generated using the coef and confint function within the stats package in R. Covariates included in the model were selected based on previously reported effects on NAb responses after two or three doses of COVID-19 vaccine. The reference was chosen for covariates with multiple categories to reflect the group with the least expected effect on NAb response. Anti-CD20 and BTKi treatments were combined in a single covariate based on their similar effect on B cell levels. Other treatments were combined to a single variable based on previous experience of their limited impact.[1,4,30]

ADDITIONAL RESOURCES

Clinical trial registry number: NCT0322688.

Cell Reports Medicine, Volume *3*

Supplemental information

Functional immune responses against SARS-CoV-2 variants of concern after fourth COVID-19 vaccine dose or infection in patients with blood cancer

Annika Fendler, Scott T.C. Shepherd, Lewis Au, Mary Wu, Ruth Harvey, Katalin A. Wilkinson, Andreas M. Schmitt, Zayd Tippu, Benjamin Shum, Sheima Farag, Aljosja Rogiers, Eleanor Carlyle, Kim Edmonds, Lyra Del Rosario, Karla Lingard, Mary Mangwende, Lucy Holt, Hamid Ahmod, Justine Korteweg, Tara Foley, Taja Barber, Andrea Emslie-Henry, Niamh Caulfield-Lynch, Fiona Byrne, Daqi Deng, Svend Kjaer, Ok-Ryul Song, Christophe J. Queval, Caitlin Kavanagh, Emma C. Wall, Edward J. Carr, Simon Caidan, Mike Gavrielides, James I. MacRae, Gavin Kelly, Kema Peat, Denise Kelly, Aida Murra, Kayleigh Kelly, Molly O'Flaherty, Robyn L. Shea, Gail Gardner, Darren Murray, Sanjay Popat, Nadia Yousaf, Shaman Jhanji, Kate Tatham, David Cunningham, Nicholas Van As, Kate Young, Andrew J.S. Furness, Lisa Pickering, Rupert Beale, Charles Swanton, Sonia Gandhi, Steve Gamblin, David L.V. Bauer, George Kassiotis, Michael Howell, Emma Nicholson, Susanna Walker, Robert J. Wilkinson, James Larkin, and Samra Turajlic

Table S1: Ordinal regression model of factors associated with Omicron BA.1 NAbT responses after 4 vaccine doses

	Ordinal regression model				ANOVA	
Factor	Coefficient	S.E.	Wald Z	P-value	Chi-Square	p-value
**Vaccine type (first and second dose)**					0.13	0.72
BNT162b2 (vs ChAdOx1)	-0.17	0.48	-0.35	0.72		
**Previous infection**					3.44	0.06
SarS-CoV-2 infection before second vaccine dose	1.40	0.76	1.86	0.06		
**Diagnosis (vs acute leukaemia)**					3.52	0.47
Chronic lymphocytic leukaemia	0.34	0.98	0.35	0.72		
Myelodysplastic syndrome	-0.95	1.41	-0.67	0.50		
Myeloma	0.11	0.98	0.12	0.91		
Lymphoma	1.31	1.04	1.26	0.21		
**Anti-cancer therapy**						
B cell-depleting therapy	-3.72	0.85	-4.35	<0.0001	18.89	<.0001
Chemo- or targeted therapy	-0.15	0.64	-0.24	0.81	0.06	0.81

NAbT were binned in undetected/low (=< 40), moderate (>40-256), or high (>256) †For anti-cancer therapy indicated treatment was tested for patients who received the treatment vs patients not receiving that treatment. BTKi, Bruton's tyrosine kinase inhibitor.

Table S2: Ordinal regression model of factors associated with Omicron BA.2 NAbT responses after 4 vaccine doses

	Ordinal regression model				ANOVA	
Factor	Coefficient	S.E.	Wald Z	P-value	Chi-Square	p-value
**Vaccine type (first and second dose)**					0.00	0.98
BNT162b2 (vs ChAdOx1)	-0.01	0.51	-0.02	0.98		
**Previous infection**					1.01	0.32
SarS-CoV-2 infection before second vaccine dose	0.76	0.76	1.00	0.32		
**Diagnosis (vs acute leukaemia)**					3.5	0.48
Chronic lymphocytic leukaemia	0.85	1.00	0.85	0.40		
Myelodysplastic syndrome	1.17	1.55	0.75	0.45		
Myeloma	1.52	1.04	1.46	0.14		
Lymphoma	1.57	1.06	1.48	0.14		
**Anti-cancer therapy**						
B cell-depleting therapy	-3.01	0.81	-3.73	0.0002	13.88	0.0002
Chemo- or targeted therapy	-0.62	0.73	-0.85	0.40	0.72	0.40

NAbT were binned in undetected/low (=< 40), moderate (>40-256), or high (>256) †For anti-cancer therapy indicated treatment was tested for patients who received the treatment vs patients not receiving that treatment. BTKi, Bruton's tyrosine kinase inhibitor.
